# Bold or reckless? The impact of workplace risk-taking on attributions and expected outcomes

**DOI:** 10.1371/journal.pone.0228672

**Published:** 2020-03-04

**Authors:** Susan R. Fisk, Jon Overton

**Affiliations:** Department of Sociology, Kent State University, Kent, Ohio, United States of America; Middlesex University, UNITED KINGDOM

## Abstract

Risk-takers are rhetorically extolled in America, but does this veneration ignore the downsides of failure? We test competing perspectives on how workplace risk-takers are perceived by examining cultural attitudes about individuals who successfully take, unsuccessful take, and avoid risks at work. The results of two experiments show that, in comparison to risk-avoidance, expected workplace outcomes are enhanced by successful risk-taking and that failure does not appear to significantly harm expected workplace outcomes for risk-takers. While one experiment finds that failed risk-takers are seen as more likely to be downsized (because they are viewed as more foolish), we also find failed risk-takers are perceived as more likely to be hired and promoted. Mediation analyses reveal this is primarily because risk-taking—regardless of outcome—considerably increases perceptions of agency and decreases perceptions of indecisiveness, and these attributions predict positive workplace outcomes. We also find the results to be remarkably similar across varying participant characteristics (namely, gender, race, education level, work experience, income, and age), which suggests that there is a broad cultural consensus in the U.S. about the value of risk-taking. In sum, we find evidence that observers generally make more positive attributions about risk-takers than about risk-avoiders, even when risk-takers fail.

## Introduction

Risk-taking and risk-takers are culturally praised in the United States [1,2]. Ethnographic accounts of white-collar workplaces document that those who take risks are seen as active go-getters [3] and as having what it takes to lead effectively [4]. Successful people also frequently espouse the importance of risk-taking for career success [5,6]: for instance, Mark Zuckerberg famously asserted “The biggest risk is not taking any risk,” and Richard Branson, founder of the Virgin Group, has been quoted as saying that, “Every risk is worth taking as long as it’s for a good cause, and contributes to a good life.” But is this veneration of risk-takers simply a form of survivorship bias that overlooks the impact of failure on attributions?

The expansive literature on negativity bias, loss aversion, and attributions would answer a resounding, “Yes.” Research on negativity bias and risk aversion finds that people pay disproportionate attention to negative stimuli, such as failure [7], suggesting that observers would perceive failed risk-taking quite negatively. Moreover, the material rewards associated with risk-taking should, in and of themselves, influence judgements about risk-takers, as experimental research has found that when people become arbitrarily associated with rewards or success, people judge them more favorably than those associated with fewer rewards or failure [8,9]. This suggests that outcomes of risk-taking behavior will influence attributions made by observers, with successful risk-takers being perceived as more competent (given that they are more likely to receive material rewards), and failed risk-takers as less competent (given that they are less likely to receive material rewards), than risk-avoiders. Since perceived competence guides the distribution of workplace and reward outcomes generally [10], these attributions should further magnify the benefits of successful risk-taking and amplify the costs of unsuccessful risk-taking. Thus, this “negativity bias perspective,” strongly suggests that, relative to risk-avoiders, risk-takers’ reputations are likely to face more downside from failed risk-taking than upside from successful risk-taking.

However, failed workplace risk-taking may have fewer negative effects on attributions than the negativity bias perspective would predict. Through its intimate connection with entrepreneurialism [11], risk-taking signals a plucky agency and confidence [3]. Many ethnographic accounts of workplaces also testify to the social value attached to risk-taking, from the managerial world [4] to bond sales [12] to the tech sector [3]. Even when risk-takers did not succeed, Neff (2012) explained that the mere act of taking risks became seen by certain tech workers as signaling autonomy and independent thought, in contrast to passive “worker-bee” employees. These anecdotes highlight that although failure is aversive, just the act of taking a risk can signal that a person is agentic and motivated. Moreover, risk-taking can signal confidence, a socially valued trait [13]. In an extreme demonstration, Kennedy, Anderson, and Moore [14] found that after displaying overconfident behavior, a person could retain their high rank, even after group members learned this person was less capable than their behavior suggested. Given this, the act of taking a risk at work—independent of outcome—could cause risk-takers to be perceived more positively by observers than risk-avoiders. These positive perceptions could, in turn, produce positive workplace outcomes even in the event of failure.

Failed risk-taking may also have fewer negative effects on workplace outcomes than the negativity bias would predict, given that some workers are rewarded by their firms even when they take risks and fail [15]. This is because such firms want to encourage their workers to overcome their risk-aversion and take risks, as the rewards attached to successful risk-taking (like innovation) can be quite large to a firm. These rewards would also make it so that failed risk-takers could still obtain positive workplace outcomes. Moreover, given that objects and social attributes generally becomes more culturally valued as the material rewards associated with them increase, [16,17] any material gains associated with risk-taking should increase further increase the cultural value of risk-taking. Thus, this “societal value,” perspective suggests that risk-takers’ reputations may not suffer much from failed risk-taking, and that, relative to risk-avoiders, risk-takers’ reputations are likely to face more upside from successful risk-taking than downside from failed risk-taking.

In the present research, we test these competing perspectives by examining cultural attitudes about individuals who successfully take, unsuccessfully take, and avoid risks at work. Specifically, we measure how risk-taking and risk-avoidance impact observers’ perceptions of employee workplace outcomes (e.g., being fired, hired, promoted), attributions about employees (e.g., beliefs about workers’ competence, agency, likability, etc.), and how these attributions mediate the effect of risk-taking behavior on expected workplace outcomes. For the purposes of this research, we define workplace risk-taking behavior as making a choice in an occupational setting where: 1) there is uncertainty about the outcome resulting from the action, 2) random chance influences the outcome, and 3) the action can yield a better or worse outcome relative to risk-avoidance [18].

## Methodological approach

We use experimental vignette studies to assess cultural attitudes about individuals who take and avoid risks at work, as vignette studies are ideally suited for studying cultural beliefs (for recent examples, see [19,20]). In addition, the use of experimental vignettes allowed us to precisely manipulate our independent variable of interest (i.e., risk-taking) in a manner that would be difficult to control in an actual workplace, thereby eliminating confounders. Although vignette studies cannot provide information on actual employee outcomes, they allow us to understand how risk-takers and risk-taking behavior are understood by observers.

Our vignettes have participants compare a risk-taking employee against a risk-avoiding employee. This allows us to more fully ascertain the impact of risk-taking on perceptions and attributions about employees, as it allows us to compute the effect of risk-taking relative to the alternative course of behavior (i.e., not taking a risk). All vignettes described two employees who both had the opportunity to engage in a workplace behavior that could be understood as risk-taking, as the behavior was described as having both more upside (in the event of success) and more downside (in the event of failure) than not engaging in the behavior. For instance, one vignette scenario involves two employees being asked if they would like to lead a new initiative at work. Assuming a leadership role constitutes taking a risk at work, because a successful leader will have a successful project on their record that in some way benefitted their organization (i.e., upside potential), while a failure will be seen as a harm to the organization and potentially stain the failed leader’s record (i.e., downside potential). This is in comparison to an individual who *avoids* assuming a leadership role, and who has no possibility of being either a successful or unsuccessful leader (i.e., no upside or downside potential).

If the “negativity bias,” perspective is accurate, we would expect that successful risk-takers would receive an advantage (in regard to expected workplace outcomes, perceptions, and attributions) relative to risk-avoiders. But we would expect the absolute value of this advantage to be *smaller* than the absolute value of the disadvantage conferred to failed risk-takers. In other words, we would expect failed risk-taking to hurt more than successful risk-taking helps (relative to risk-avoidance). On the other hand, if the, “societal value,” perspective is accurate, we would expect that successful risk-takers would receive an advantage (in regard to expected workplace outcomes, perceptions, and attributions) relative to risk-avoiders. In this case, the absolute value of the advantage would be *larger* than the absolute value of the disadvantage conferred to failed risk-takers (i.e., more upside than downside potential relative to risk avoidance).

Both Study 1 and Study 2 were approved by Stanford’s Institutional Review Board (IRB).

## Study 1—Risk-taking and workplace outcomes

### Study 1 methods

#### Design and procedure

The goal of Study 1 was to learn how observers believed workplace risk-taking would impact workplace outcomes. Participants read a vignette that described two employees who had the opportunity to take a risk at work. In the vignette, one employee (the risk-taker) always accepted the opportunity, and one employee (the risk-avoider) always rejected the opportunity. Participants then made comparative judgements about the employees (for instance, which employee—the risk-taker or the risk-avoider—they thought was more likely to be downsized). The gender of the risk-taking and risk-avoiding employee was randomly assigned. While we found some instances of a woman risk-taker receiving slightly greater advantages relative to a man risk-taker, these differences were slight and inconsistent. Thus, the analyses do not separate workplace risk-takers and risk-avoiders on the basis of gender.

Participants read one of six vignettes that varied the outcome for the risk-taker (success or failure) and the workplace scenario (offering a suggestion at a meeting, innovating a workplace procedure, or leading a new initiative). The vignettes used different types of workplace scenarios to ensure results were driven by risk-taking—the theoretical concept of interest—and not tangential particularities of any specific situation. See Table 1 for an illustration of the six vignette scenarios and S1 Data, Part A for the full wording of the vignettes.

**Table 1:** Overview of vignettes.

		**Workplace Scenario**		
		*Suggestion at meeting*	*Innovating a workplace procedure*	*Leading a new initiative*
**Comparison**	*Successful risk-taker vs*. *risk-avoider*	Vignette 1	Vignette 3	Vignette 5
	*Failed risk-taker vs*. *risk-avoider*	Vignette 2	Vignette 4	Vignette 6

Once participants provided informed consent, they completed a brief attention check to ensure they understood the study and its purpose. Participants then read the scenarios, after which they were asked to write about their independent impressions of the risk-taking employee, their independent impressions of the risk-avoiding employee, and then their impression of the risk-taking employee relative to the risk-avoiding employee. This exercise was designed to engage online participants in the study and to force them to think deeply about the employees and their behavior. Participants were then asked a series of questions about the workplace outcomes likely to occur for the risk-taking employee relative to the risk-avoiding employee (see below for details). To conclude the study, participants answered several questions about their own work history, demographics, and study experiences.

#### Workplace outcome measures

We measured five different workplace outcomes. Three were rank outcomes (downsizing, promotion, being sent to a, “special management training program for employees with high leadership potential,”) and two were distribution outcomes (pay, bonus). For the outcomes related to rank, participants were asked to give the odds that the event (e.g., being downsized, promoted, or sent to high potential leadership training camp) would happen to the risk-taking versus risk-avoiding employee. Participants then distributed a fixed pool of 100 percentage points between the two employees. If a participant thought that there were equal odds that either the risk-taking or risk-avoiding employee would be downsized, the participant would put a 50% likelihood on each employee. For the distribution outcomes, participants were asked how much money they thought each employee currently earned and how the company would distribute a fixed bonus between the two employees. For interpretative ease, the income variable was recoded into a percentage of the maximum possible salary and the bonus recoded to the percentage of the total bonus.

#### Participants

Participants were recruited from Amazon Mechanical Turk (mTurk). MTurk is an online marketplace in which a national sample of adults (known as mTurkers) are paid to complete online tasks that humans can perform more effectively than computers (e.g., identifying objects in a photo). While mTurk provides a convenience sample, it does contain workers from many different walks of life, and thus is much more representative of the population than most in-person convenience samples (especially those from college campuses) [21,22].

The study was advertised on mTurk as a survey about workplace experiences. Participants were paid $1.50 for approximately 15 minutes of work. Three hundred and twenty-two mTurk workers participated in the study, of whom 60.1% were women and 78.2% were non-Hispanic white. Participants ranged in age from 18 to 75 years, with an average age of 32.8 years and a standard deviation of 10.1 years. Of all participants in Study 1, 46.6% were between 18–29 years old, 31.9% were between 30–39 years old, 13.5% were between 40–49 years old, 5.8% were between 50–59 years old, and 2.2% were over 60 years old. 11.4% of the sample was unemployed, and 66.3% of the sample had attended at least some college.

### Study 1 results

All Study 1 results are calculated using two-tailed t-tests comparing the mean value (from all vignette scenarios) against a value of 50%. Answering with a value of 50% means a respondent thought that the risk-taker and risk-avoider had equal odds of experiencing an event. Therefore, our t-tests test the null hypothesis that there was no difference in the rating of the risk-taker relative to the risk-avoider.

#### Workplace outcomes for successful risk-takers relative to risk-avoiders

Across all scenarios and all measures of workplace outcomes, successful risk-takers were rated as much more likely to have positive workplace outcomes (e.g., being promoted, sent to high potential leadership training camp) and much less likely to have negative workplace outcomes (e.g., being downsized) than risk-avoiders. We find that successful risk-takers are rated as: much less likely to be downsized (27.4%, *p* < .001), much more likely to be promoted (79.0%, *p* < .001), and much more likely to be sent to a leadership training camp for high-potential employees (78.8%, *p* < .001) than not (Table 2). In addition, respondents believed that successful risk-takers would receive a higher amount of total possible pay than risk-avoiders (53.0%, *p* < .001) and of the bonus (72.1%, *p* < .001). Interestingly, these effects did not vary greatly between scenarios.

**Table 2:** Study 1 t-tests of ratings of workplace outcomes (risk-taking versus risk-avoiding employees) (N = 322).

	*Overall*			*Meeting*			*Leading Initiative*			*Job Innovation*		
	*M*	*SD*	*N*	*M*	*SD*	*N*	*M*	*SD*	*N*	*M*	*SD*	*N*
**Successful Risk-Taker**													
Downsize	27.4***	16.8	158	25.1***	15.5	54	28.0***	18.8	52	29.3***	16.1	52
Pay	53.0***	4.0	158	53.3***	4.8	54	53.3***	3.4	52	52.5***	3.7	52
Bonus	72.1***	14.0	158	69.4***	15.0	54	76.3***	13.3	52	70.7***	12.7	52
Promote	79.0***	13.8	158	77.5***	16.1	54	81.0***	12.9	52	78.7***	12.2	52
Leadership Training	78.8***	17.7	158	79.4***	16.2	54	77.8***	18.5	52	79.2***	18.6	52
**Failed Risk-Taker**													
Downsize	54.5**	18.6	164	49.5	16.8	54	60.8***	18.0	54	53.4	19.3	56
Pay	50.8**	3.3	164	50.9	4.1	54	50.8*	2.8	54	50.6	3.7	56
Bonus	51.2	1.4	164	58.2***	14.3	54	45.6	19.0	54	49.8	19.0	56
Promote	55.7***	19.5	164	59.5**	20.1	54	51.6	17.1	54	55.9*	20.6	56
Leadership Training	65.1***	20.9	164	67.1***	18.8	54	60.3**	21.9	54	67.8	21.5	56

*p < .05.

**p < .01.

***p < .001.

#### Workplace outcomes for failed risk-takers relative to risk-avoiders

The effect of failed risk-taking on workplace outcomes was more mixed, as failed risk-taking was seen as more likely to produce negative outcomes (e.g., being downsized), as having no meaningful effect on the distribution of monetary resources, and having a positive effect on the likelihood of experiencing positive outcomes (e.g., being promoted, sent to high potential leadership training camp) relative to risk-avoidance. We find that failed risk-takers are rated as more likely to be downsized (54.5%, *p* < .01) than risk-avoiders (Table 2). However, this effect varies by scenario and appears to correspond to the severity of failure: individuals who fail at leading an initiative are seen as more likely to be downsized (60.8%, *p* < .001) than the risk-avoiders, while it does not appear that failed innovation or poor suggestions at meetings have a statistically significant impact on the likelihood of being downsized (Table 2).

Second, we do not find evidence that failed risk-takers are disadvantaged in the expected distribution of monetary resources. Failed risk-takers are perceived as having a slightly higher proportion of pay than risk-avoiders (average proportion of 50.7% versus 49.3%, *p* < .01) and there are no statistically significant differences in the expected bonus amount (Table 2).

Lastly, and perhaps most interestingly, failed risk-takers are generally *advantaged* relative to risk-avoiders in regard to promotion and leadership. We find that failed risk-takers are rated as more likely to be promoted (55.7%, *p* < .001) and sent to high-potential leadership training camp (65.1%, *p* < .001) than risk-avoiders (Table 2).

## Study 2—Risk-taking, workplace outcomes, and attributions

### Study 2 methods

#### Design and procedure

The goal of Study 2 was to understand how risk-taking and risk-avoidance influence the attributions made about employees. We did this in hopes of discerning a puzzling result in Study 1: why does failed risk-taking behavior *not* have a profoundly negative impact on expected workplace outcomes? Research on negativity bias gives us every reason to expect the opposite of what we found. To do this, we repeated the procedures used in Study 1. We then asked respondents to give independent trait ratings about the risk-taking and risk-avoiding employees using 100-point slider scales (in which 0 = “Not at all,” and 100 = “Completely”). These trait ratings were combined to create six indexes: likability, dominance, agency, indecisiveness, competence, and foolishness. Participants also independently rated each employee using these slider bars on the workplace outcomes of downsizing, interview, and promotion. See S2 Data, Part B for details on items and scale reliability, Table 3 for summary statistics, and Table 4 for a correlation matrix.

**Table 3:** Descriptive statistics for study 2 (N = 1,110 observations).

Variable	Mean / Proportion	Standard Deviation	Range
Successful Risk-Taker	0.27	–	0–1
Failed Risk-Taker	0.24	–	0–1
Indecisiveness	31.95	26.00	0–100
Foolishness	28.40	22.46	0–100
Dominant	30.37	22.86	0–100
Competent	61.29	23.38	0–100
Agentic	52.45	29.62	0–100
Likable	53.52	21.79	0–100
Promote	52.32	28.63	0–100
Interview	58.81	27.93	0–100
Downsize	30.59	25.73	0–100

Standard deviations do not correct for observations clustered within individuals.

**Table 4:** Correlation matrix for study 2 trait attributions and workplace outcomes (N = 1,110 observations).

	(1)	(2)	(3)	(4)	(5)	(6)	(7)	(8)	(9)	(10)	(11)
(1) Successful Risk-Taker	1.00										
(2) Failed Risk-Taker	-1.00***	1.00									
(3) Indecisiveness	-.06*	.06*	1.00								
(4) Foolishness	-.21***	.21***	.47***	1.00							
(5) Dominant	-.06*	.06*	-.20***	.28***	1.00						
(6) Competent	.20***	-.20***	-.41***	-.46***	.21***	1.00					
(7) Agentic	.03	-.03	-.66***	-.19***	.58***	.60***	1.00				
(8) Likable	.12***	-.12***	-.29***	-.27***	.22***	.76***	.53***	1.00			
(9) Promote	.15***	-.15***	-.53***	-.37***	.35***	.78***	.76***	.69***	1.00		
(10) Interview	-.18***	.18***	.54***	.58***	.02	-.48***	-.43***	-.33***	-.51***	1.00	
(11) Downsize	.12***	-.12***	-.47***	-.38***	.30***	.80***	.70***	.75***	.83***	-.52***	1.00

*p < .05.

**p < .01.

***p < .001.

Correlation coefficients do not correct for observations clustered within individuals.

#### Participants

Participants were again recruited from Amazon Mechanical Turk, and the study was again advertised as a survey about workplace experiences. Participants were paid $2.00 for approximately 20 minutes of work. Five hundred and fifty-five Amazon Mechanical Turk workers participated in the study, of which 54.2% were women and 82.2% were white. Participants ranged in age from 18 to 73 years, with an average age of 32.7 years and a standard deviation of 10.2 years. 46.8% were between 18–29 years old, 32.2% were between 30–39 years old, 13.1% were between 40–49 years old, 5.6% were between 50–59 years old, and 2.2% were between 60–69 years old. 14.2% of the sample was unemployed, and 69.6% of the sample had attended at least some college.

### Study 2 results

#### Workplace outcomes for risk-takers relative to risk-avoiders

Our Study 2 results replicate those from Study 1 with one exception: in Study 2, we found no overall difference across scenarios between the failed risk-taker and the risk-avoider in the perceived likelihood of being downsized. However, this does vary by scenario, as risk-takers were seen as more likely to be downsized than a risk-avoider if they failed at leading an initiative (57.1%, *p* < .001), but less likely to be downsized than a risk-avoider if they offered a poor idea at a meeting (42.8%, *p* < .001). See the t-tests in Table 5 for details.

**Table 5:** Study 2 t-tests of ratings of workplace outcomes (N = 555).

	*Overall*			*Meeting*			*Leading Initiative*			*Job Innovation*		
	*M*	*SD*	*N*	*M*	*SD*	*N*	*M*	*SD*	*N*	*M*	*SD*	*N*
**Successful Risk-Taker**												
Downsize	23.6***	17.7	269	27.5***	19.7	94	18.9***	17.9	78	23.6***	14.4	97
Pay	52.2***	2.8	269	52.3***	3.1	94	53.1***	2.8	78	51.4***	2.3	97
Bonus	73.2***	17.1	269	69.9***	18.5	94	78.5***	14.0	78	72.2***	17.0	97
Promote	77.9***	17.0	269	75.3***	18.6	94	84.1***	14.7	78	75.5***	16.1	97
Leadership Training	76.0***	22.3	269	73.2***	24.1	94	79.5***	22.9	78	76.0***	2.0	97
**Failed Risk-Taker**												
Downsize	49.7	22.6	286	42.8***	1.8	113	57.1***	21.5	73	52.0	25.3	100
Pay	50.5***	2.4	286	50.9***	2.5	113	50.1	2.7	73	50.4*	1.9	100
Bonus	49.7	21.2	286	54.6**	18.7	113	45.8	22.4	73	46.9	22.1	100
Promote	53.2*	21.4	286	57.6***	16.9	113	46.7	22.1	73	53.0	24.3	100
Leadership Training	61.4***	23.2	286	64.9***	18.2	113	56.9*	26.9	73	60.7***	24.8	100

*p* < .05.

**p < .01.

***p < .001.

#### Effect of risk-taking and risk-avoidance on trait ratings

We next determined the effect of risk-taking on trait ratings, using linear mixed models (presented in Table 6), which are multilevel models that account for lack of independence between observations. These models were necessary, as observations were nested within participant, as each participant contributed two sets of trait ratings (one for the risk-avoider and one for the risk-taker). Thus, the unit of analysis is the rating of the target employee. All traits were rated on a 100-point scale, and the coefficients represent the effect of successful and failed risk-taking relative to risk-avoidance on trait ratings.

**Table 6:** Study 2 multilevel mixed models predicting trait ratings (N = 1,110 observations nested within 555 participants).

	*Likability*	*Dominance*	*Agency*	*Indecisiveness*	*Competence*	*Foolishness*
	Coef. (Robust SE)	Coef. (Robust SE)	Coef. (Robust SE)	Coef. (Robust SE)	Coef. (Robust SE)	Coef. (Robust SE)
Failed Risk-Taker	5.4*** (1.3)	23.8*** (1.3)	42.9*** (1.4)	-29.4*** (1.4)	3.1* (1.4)	8.9*** (1.5)
Successful Risk-Taker	13.2*** (1.3)	21.7*** (1.3)	47.8*** (1.4)	-36.7*** (1.5)	21.0*** (1.4)	-13.8*** (1.6)
Intercept	48.8*** (0.9)	19.2*** (0.9)	29.5*** (0.8)	48.9*** (0.8)	55.1*** (0.9)	29.7*** (0.9)

Reference category is risk-avoidant employee.

*p* < .05.

**p < .01.

***p < .001.

Standard errors appear in parentheses.

In comparison to risk-avoiders, we find that risk-takers are seen as slightly more likable (failed risk-taker: *b* = 5.4, *p* < .001, successful risk-taker: *b* = 13.2, *p* < .001), more dominant (failed risk-taker: *b* = 23.8, *p* < .001, successful risk-taker: *b* = 21.7, *p* < .001), much more agentic (failed risk-taker: *b* = 42.9, *p* < .001, successful risk-taker: *b* = 47.9, *p* < .001), and much less indecisive (failed risk-taker: *b* = -29.4, *p* < .001, successful risk-taker: *b* = -36.7, *p* < .001) than risk-avoiders. We also find that risk-takers are seen as more competent than risk-avoiders, although the magnitude of this effect varies greatly by whether the risk was taken successfully or not (failed risk-taker: *b* = 3.1, *p* < .05, successful risk-taker: *b* = 21.0, *p* < .001). Lastly, we find that foolishness is the only trait that disadvantages failed risk-takers: while successful risk-takers are seen as less foolish (*b* = -13.8, *p* < .001), failed risk-takers are seen as more foolish (*b* = 8.9, *p* < .001) than risk avoiders.

#### Impact of trait ratings on workplace outcomes

We again use linear mixed models to determine the impact of these trait ratings on workplace outcomes. These models account for lack of independence between observations, as the unit of analysis was the employee (Table 7). The dependent variables were the independent, 100-point ratings of each employee on the following workplace outcomes: downsizing, interview, and promotion.

**Table 7:** Study 2 multilevel mixed models predicting effect of trait ratings on workplace outcomes (N = 1,110 observations nested within 555 participants).

	*Downsize*	*Interview*	*Promote*
	Coef. (Robust SE)	Coef. (Robust SE)	Coef. (Robust SE)
Likability	0.04 (.04)	0.38*** (.03)	0.24*** (.03)
Dominance	0.12*** (.03)	0.00 (.02)	0.01 (.03)
Agency	-0.18*** (.04)	0.30*** (.03)	0.42*** (.03)
			
Indecisiveness	0.21*** (0.03)	0.00 (.02)	-0.01 (.03)
Competence	-0.21*** 0.05	0.42*** (.03)	0.42*** (.03)
Foolishness	0.38*** (.04)	-0.09*** (.03)	-0.10*** (.03)
Intercept	29.39*** (2.67)	-0.58 (1.90)	-5.49** (1.93)

Standard errors appear in parentheses.

*p* < .05.

**p < .01.

***p < .001.

We find two general trends in the data. First, some traits appear to have larger and more consistent impacts on workplace outcomes than others. All workplace outcomes were significantly impacted by agency, competence, and to a lesser degree, foolishness. On the other hand, dominance and indecisiveness only predicted the likelihood that an employee would be downsized, and the magnitude of these effects was relatively small. Second, we find that negative traits (i.e., dominance, indecisiveness, foolishness) are typically more predictive of negative outcomes (i.e., being downsized), while positive traits (i.e., likability, agency, and competence) are more predictive of positive workplace outcomes (i.e., being interviewed or promoted).

#### Mediation analyses

To construct a model by which risk-taking affects workplace outcomes through trait attributions, we conducted a series of structural equation models. Our exogenous variables are two dummy variables: whether risk-taking succeeded or failed. This makes the risk-avoiding employee the reference category. Standard errors were correlated in these models, to correct for the non-independence of observations.

**Fig 1:**
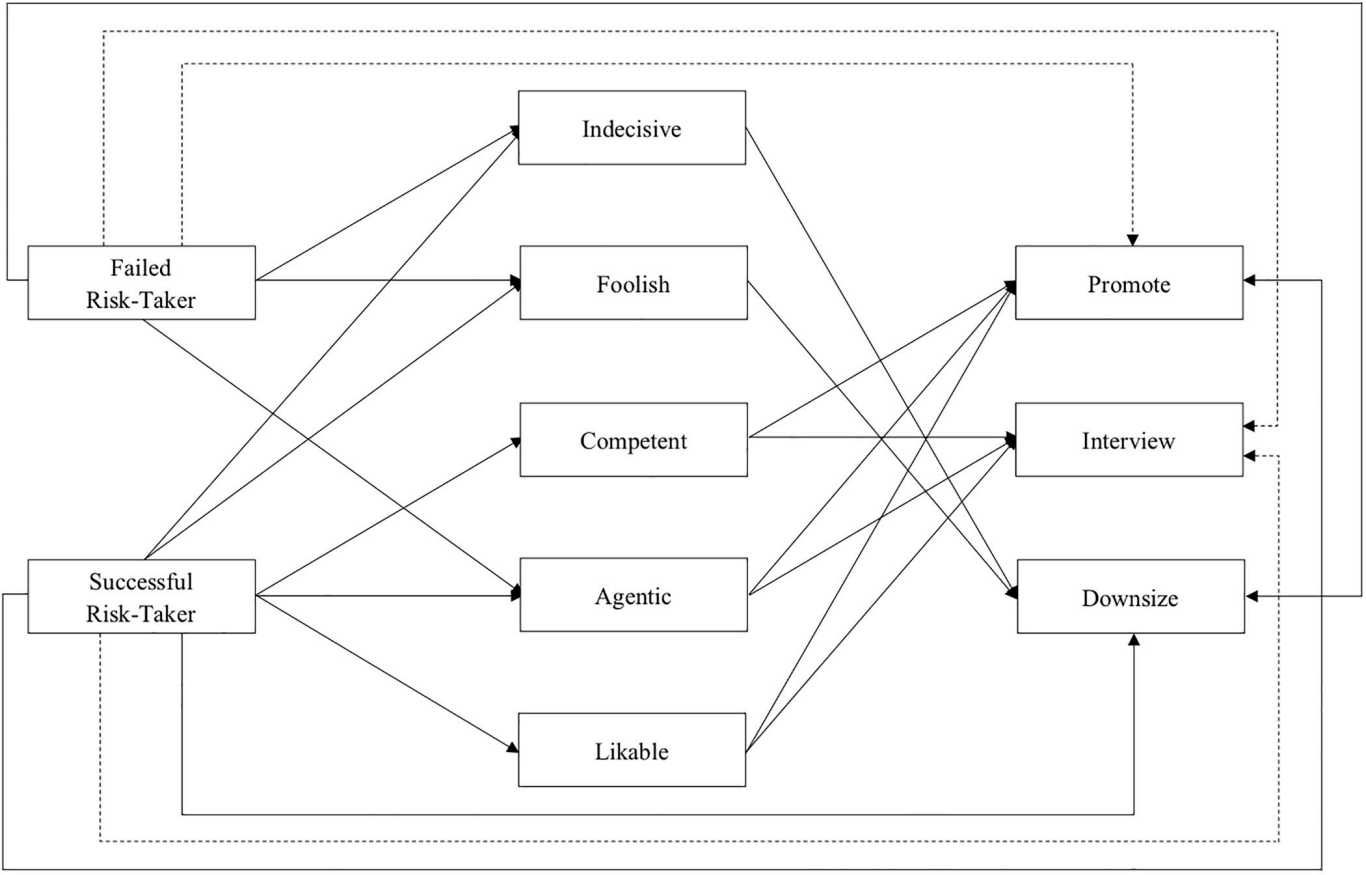
Path model depicting the effect of risk-talking relative to risk-avoidance on trait attributions and workplace outcome (n = 1,110 observations clustered within 555 individuals). *NOTE*: Solid lines represent statistically paths (*p* < .05). Dashed lines represent non-significant paths.

The unrefined model used failed and successful risk-taking (where the risk-avoider is the reference category) to predict all measured trait attributions and work outcomes. Then all trait attributions predicted all workplace outcomes (see S1 Table). Given that was a poorly fitting model (*χ*^2^ [18] = 2,066.02, *p* < .001, RMSEA = .320, CFI = .679, SRMR = .181), and following the standard model construction steps for structural equation and path models (outlined by Kline [23]), we continually respecified the model by removing paths that were not statistically significant (i.e., paths that although plausible, effectively do not exist) and allowing endogenous variables to covary that were creating substantial residuals. The refined model, which drops the dominance attribution and a number of paths, is presented in Table 8 and seen in Fig 1. The model’s predicted values are significantly different from the observed values (*χ*^2^ [9] = 46.77, *p* < .001), but the goodness-of-fit statistics are otherwise good (CFI = .993; RMSEA = .061; SRMR = .026).

**Table 8:** Refined path model coefficients predicting trait attributions and workplace rewards (N = 1,110 observations nested within 555 participants).

	*Trait Attributions*					*Workplace Rewards*		
	Indecisive	Foolish	Competent	Agentic	Likable	Promote	Interview	Downsize
	Coef. (Robust SE)	Coef. (Robust SE)	Coef. (Robust SE)	Coef. (Robust SE)	Coef. (Robust SE)	Coef. (Robust SE)	Coef. (Robust SE)	Coef. (Robust SE)
Successful Risk-Taking	-36.51*** (1.32)	-13.48*** (1.39)	20.00*** (1.36)	46.98*** (1.40)	11.71*** (1.36)	7.92*** (1.68)	1.83 (1.50)	-6.84*** (1.74)
Failed Risk-Taking	-28.75*** (1.31)	9.72*** (1.42)		40.52*** (1.19)		.60 (1.66)	1.33 (1.55)	-4.69*** (1.79)
Indecisive								.22*** (.04)
Foolish								.41*** (.04)
Competent						.45*** (.04)	.48*** (.04)	-.22*** (.04)
Agentic						.37*** (.04)	.26*** (.04)	
Likable						.25*** (.04)	.37*** (.04)	
Constant	48.64*** (.95)	29.44*** (.86)	56.12*** (.85)	30.34*** (.84)	50.52*** (.82)	-10.49*** (1.09)	-5.30 (1.23)	28.30*** (3.36)

Fit statistics: *χ*^*2*^ (9) = 46.77, *p* < .001; CFI = .993; TLI = .967; RMSEA = .061; SRMR = .026.

*p < .05.

**p < .01.

***p < .001.

Standard errors appear in parentheses.

We find that successful risk-taking positively influences workplace outcomes both indirectly (through its beneficial impact on measures of agency, competence, and likability) and directly. By increasing ratings of agency, competence, and likability, successful risk-taking increases the odds of receiving an interview (indirect effect = 26.15, *p* < .001) and a promotion (indirect effect = 29.31, *p* < .001). The total effect of successful risk-taking on receiving an interview can be broken down as follows: 43.7% indirect effect through agency, 34.3% indirect effect through competence, 15.5% indirect effect through likability, and 6.5% direct effect. The total effect of successful risk-taking on receiving a promotion can be broken down as follows: 46.7% indirect effect through agency, 24.2% indirect effect through competence, 7.9% indirect effect through likability, and 21.3% direct effect (Table 9). Notably, the effect of successful risk-taking on trait attributions account for 93.5% of the effect of successful risk-taking on getting a job interview and 79.7% of the effect for getting a promotion.

**Table 9:** Percentages of total effects on workplace rewards accounted for by trait attributions (N = 1,110 observations nested within 555 participants).

	Successful Risk-Taking	Failed Risk-Taking
Percent of Downsize Effect		
Indecisive	32.4%	42.2%
Competent	17.7%	No Path
Foolish	22.3%	26.6%
Risk-Taking (Remainder)	27.6%	31.3%
Percent of Promotion Effect		
Agentic	46.7%	96.2%
Likable	7.9%	No Path
Competent	24.2%	No Path
Risk-Taking (Remainder)	21.3%	3.8%
Percent of Interview Effect		
Agentic	43.7%	88.8%
Likable	15.5%	No Path
Competent	34.3%	No Path
Risk-Taking (Remainder)	6.5%	11.2%

Through reducing perceptions of foolishness and indecision, successful risk-taking also decreases the odds of being downsized (indirect effect = 17.96, *p* < .001). The total effect of successful risk-taking on being downsized can be broken down as follows: 32.4% indirect effect through indecisiveness, 22.3% indirect effect through foolishness, 17.7% indirect effect through competence, and 27.6% direct effect (Table 9). Thus, while we find evidence that successful risk-taking—in and of itself—influences workplace outcomes, most of its effects on workplace outcomes are caused by the indirect effects of successful risk-taking on trait attributions.

We also find that failed risk-taking impacts workplace outcomes both indirectly (through its effects on attributions about agency, indecisiveness, and foolishness) and directly. By increasing perceptions of agency relative to the risk-avoidant employee (b = 40.52, *p* < .001), failed risk-taking increases the expected likelihood of receiving a promotion (indirect effect = 14.99, *p* < .001) and a job interview (indirect effect = 10.53, *p* < .001). In both cases, the indirect effect of failed risk-taking on agency accounts for nearly the entire effect of failed risk-taking on getting a promotion (96.2% indirect effect through agency, 3.8% direct effect of unsuccessful risk-taking) or a job interview (88.8% indirect effect through agency, 11.2% direct effect of unsuccessful risk-taking) (see Table 9).

Surprisingly, failed risk-takers were seen as slightly less likely to be downsized than risk-avoiders in this mediation analysis (b = -4.69, *p* < .001). This is because risk-avoidant workers were more likely to appear indecisive than risk-taking employees, even though failed-risk-takers were perceived as more foolish. The total effect of failed risk-taking on being downsized can be broken down as follows: 42.2% indirect effect through indecisiveness, 26.6% indirect effect through foolishness, and 31.3% direct effect (Table 9). Given that the direct effect of failed risk-taking on being downsized is negative, failed risk takers are perceived as less likely to be downsized than risk-avoiders even when the trait attributions are taken into account. This provides further evidence of the societal value of risk-taking, as risk-taking appears to be valued even in cases when it leads to failure.

## Supplementary analysis: Cultural consensus about the value of risk-taking

Our results from Study 1 and 2 provide evidence that risk-taking—regardless of outcome—predicts positive workplace outcomes because risk-taking increases perceptions of agency and decreases perceptions of indecisiveness. However, there could be systematic variation in how participants perceive risk-taking (based on their work experience and demographic categories). There is reason to believe that this could occur, as past research finds those with more social advantages tend to endorse values like achievement and self-direction, whereas those lower on society’s totem pole favor obedience and tradition [24–26]. Consequently, we might reasonably expect groups like men, whites, and those from higher socioeconomic backgrounds to put an especially high premium on workplace risk-taking, relative to women, minorities, and those from lower socioeconomic backgrounds. If this were to be the case, it would provide evidence against the assertation that there is a cultural consensus about the value of risk-taking in workplaces.

Thus, we conducted supplementary analyses in which we separated our analyses by participant demographics, to determine the degree of cultural agreement about the value of workplace risk-taking. In these supplementary analyses, we ran our t-tests and linear mixed models separately by gender, race, education, age, income, whether the respondent had hiring experience, and whether the respondent had managerial experience. Gender was analyzed using men and women. Nonbinary participants were necessarily excluded due to their small numbers (*N* = 2, Study 1; *N* = 3, Study 2). Race was dichotomized as non-Hispanic white vs. nonwhite. More fine-grained racial categories like blacks and Asians were too small to reliably analyze alone. Education was measured based on whether the respondent had attended college. Age was broken into three categories: 18—29, 30—39, and 40+, again driven by considerations of available statistical power. Personal income was coded into three groups: below median income (< $25,000), approximately median income ($25,000—$34,999), and above-median income ($35,000+). The median income for an individual when this data was collected was approximately $30,000 / year [27]. Participants answered yes/no questions to indicate 1) whether they had managerial experience and 2) whether they had been responsible for hiring decisions.

In Table 10, we present our one-sample t-tests from both Study 1 and Study 2 by demographic categories. Scanning these columns reveals remarkable consistency in the effects of successful and failed risk-taking on expected workplace reward outcomes. Where there is variation between demographic groups and studies, it rarely exceeds 10 points on the 100-point scale. Indeed, there is not a single demographic category that provides evidence for the, “negativity bias perspective,” as all demographic groups appear to believe that relative to risk-avoidance, there is more upside for successful risk-taking than downside for unsuccessful risk-taking.

**Table 10:** t-tests of ratings of workplace outcomes by demographic categories (pooled scenarios).

	N		Downsizing		Pay		Bonus		Promotion		Training	
Risk-Taking Outcome	Success	Fail	Success	Fail	Success	Fail	Success	Fail	Success	Fail	Success	Fail
			*M* (*SD*)	*M* (*SD*)	*M* (*SD*)	*M* (*SD*)	*M* (*SD*)	*M* (*SD*)	*M* (*SD*)	*M* (*SD*)	*M* (*SD*)	*M* (*SD*)
*All Participants*												
Study 1	158	164	27.4*** (16.8)	54.5** (18.6)	53.0*** (4.0)	50.8** (3.6)	72.1*** (14.0)	51.2 (1.4)	79.0*** (13.8)	55.7** (19.5)	78.8*** (17.7)	65.1*** (20.9)
Study 2	269	286	23.6*** (17.7)	49.7 (22.6)	52.2*** (2.8)	50.5*** (2.4)	73.2*** (17.1)	49.7 (21.2)	77.9*** (17.0)	53.2* (21.4)	76.0*** (22.3)	61.4*** (23.2)
*Gender*												
Women, Study 1	98	100	27.2*** (16.0)	56.0** (18.5)	52.3*** (3.7)	50.4 (3.6)	70.9*** (14.5)	48.3 (18.0)	78.9*** (13.6)	54.2* (19.9)	78.4*** (18.2)	64.6*** (21.5)
Women, Study 2	151	153	23.0*** (17.9)	48.6 (22.9)	52.3*** (2.7)	50.6** (2.5)	72.9*** (17.5)	49.5 (21.3)	79.0*** (17.1)	55.4** (21.7)	78.5*** (20.4)	62.9*** (23.0)
Men, Study 1	59	64	27.8*** (18.3)	52.3 (18.6)	54.3** (4.2)	51.3** (3.4)	74.1*** (12.9)	55.8* (17.7)	79.5*** (14.3)	58.0*** (18.7)	79.6*** (17.0)	65.8*** (20.1)
Men, Study 2	118	133	24.4*** (17.5)	50.9 (22.3)	52.1*** (3.0)	50.5* (2.3)	73.6*** (16.6)	49.9 (21.2)	76.6*** (17.0)	50.7 (20.9)	72.8*** (24.2)	59.6*** (23.3)
*Race*												
Whites, Study 1	124	128	27.8*** (16.2)	53.8* (18.9)	53.1*** (4.0)	50.7* (3.6)	72.2*** (14.0)	51.1 (18.2)	78.0*** (14.4)	56.7*** (19.8)	79.2*** (17.5)	65.1*** (21.3)
Whites, Study 2	202	222	24.2*** (17.7)	49.4 (22.8)	52.1*** (2.8)	50.5** (2.5)	72.7*** (17.4)	50.7 (20.8)	77.2*** (17.3)	53.6** (21.9)	75.7*** (22.4)	62.0*** (23.3)
Non-Whites, Study 1	34	36	25.9*** (19.1)	57.2* (17.2)	52.7*** (4.3)	51.2* (3.4)	71.7*** (13.9)	51.6 (18.8)	82.8*** (11.2)	52.1 (18.1)	77.5*** (18.6)	65.2*** (19.7)
Non-Whites, Study 2	67	64	21.8*** (17.7)	50.7 (21.9)	52.6*** (2.9)	50.5 (2.2)	74.7*** (16.2)	46.2 (22.4)	80.2*** (16.1)	51.8 (19.9)	76.9*** (22.3)	59.0** (22.7)
*Education*												
College, Study 1	97	113	26.5*** (15.0)	54.7* (19.1)	53.2*** (4.0)	50.2 (3.5)	73.3*** (14.5)	49.6 (18.1)	79.1*** (14.7)	54.1* (19.7)	78.4*** (18.4)	63.6*** (21.8)
College, Study 2	189	197	23.1*** (17.1)	51.1 (22.4)	52.2*** (2.8)	50.3 (2.5)	74.3*** (17.7)	47.5 (20.9)	78.6*** (17.1)	51.3 (20.3)	76.2*** (22.4)	60.9*** (23.0)
Non-College, Study 1	61	51	29.0*** (19.4)	54.3 (17.5)	52.8*** (4.0)	52.1*** (3.4)	70.2*** (12.8)	54.8 (18.2)	78.9*** (12.4)	59.1*** (18.8)	79.5*** (16.5)	68.4*** (18.5)
Non-College, Study 2	80	89	24.8*** (19.0)	46.6 (22.7)	52.3*** (2.8)	50.9*** (2.2)	70.6*** (15.4)	54.5* (21.1)	76.4*** (16.9)	57.4** (23.2)	75.6*** (22.2)	62.5*** (23.7)
*Work Experience*												
Hiring, Study 1	95	93	28.4*** (16.6)	53.1 (18.7)	53.0*** (4.2)	50.9** (3.3)	71.5*** (14.5)	50.1 (18.2)	78.7*** (13.2)	56.8** (20.5)	78.6*** (17.9)	63.8*** (22.1)
Hiring, Study 2	145	162	21.7*** (16.3)	48.9 (23.0)	52.5*** (3.0)	50.5** (2.5)	74.7*** (17.8)	50.5 (21.4)	78.6*** (17.7)	54.0* (21.6)	77.8*** (20.2)	64.1*** (22.3)
No Hiring, Study 1	63	71	26.0*** (17.2)	56.4** (18.3)	53.1*** (3.9)	50.5 (3.8)	73.0*** (13.2)	52.6 (18.3)	79.5*** (14.9)	54.1 (18.1)	79.2*** (17.5)	66.8*** (19.3)
No Hiring, Study 2	124	124	25.8*** (19.0)	50.6 (22.1)	51.9*** (2.6)	50.5* (2.3)	71.5*** (16.1)	48.6 (21.1)	77.1*** (16.2)	52.1 (21.2)	74.0*** (24.5)	57.8*** (23.9)
Management, Study 1	117	112	27.0*** (17.0)	54.5* (19.0)	53.2*** (4.1)	51.0** (3.8)	71.6*** (14.2)	51.9 (18.4)	79.8*** (13.6)	55.4** (20.5)	80.1*** (16.4)	65.1*** (21.5)
Management, Study 2	198	187	23.7*** (18.2)	50.4 (23.0)	52.4*** (3.0)	50.5** (2.4)	74.6*** (16.9)	47.9 (21.1)	78.7*** (17.0)	52.0 (22.1)	76.9*** (21.4)	61.2*** (23.2)
No Management, Study 1	41	52	28.7*** (16.3)	54.7 (17.8)	52.6*** (3.8)	50.3 (3.0)	73.4*** (13.4)	49.7 (18.1)	76.7*** (14.5)	56.2* (17.2)	75.3*** (20.8)	65.0*** (19.8)
No Management, Study 2	71	99	23.3*** (16.2)	48.3 (21.9)	51.8*** (2.3)	50.5* (2.4)	69.3*** (17.0)	53.0 (21.1)	75.8*** (17.2)	55.5** (20.0)	73.6*** (24.8)	61.6*** (23.2)
*Income*												
Below Median, Study 1	55	52	29.7*** (17.6)	55.9* (19.0)	52.7*** (4.5)	50.7 (2.9)	69.3*** (13.4)	47.4 (18.3)	76.5*** (14.3)	57.7** (19.1)	73.1*** (20.2)	63.0*** (21.2)
Below Median, Study 2	120	117	23.5*** (18.5)	50.9 (23.0)	52.0*** (2.6)	50.3 (2.5)	71.3*** (18.3)	48.8 (21.8)	78.0*** (17.8)	54.2* (22.2)	76.0*** (22.3)	60.4*** (24.5)
Median, Study 1	32	30	19.8*** (14.8)	60.5*** (15.1)	53.6*** (4.4)	51.0 (3.5)	73.7*** (14.3)	54.5 (16.3)	82.2*** (16.3)	51.0 (20.3)	88.0*** (11.3)	67.0*** (20.9)
Median, Study 2	38	43	25.0*** (18.6)	51.5 (24.6)	52.5*** (2.7)	50.7* (1.9)	73.1*** (15.6)	48.5 (24.2)	79.4*** (13.7)	52.0 (21.6)	78.5*** (21.0)	61.0** (21.6)
Above Median, Study 1	70	82	29.1*** (16.4)	51.5 (19.0)	53.2*** (3.4)	50.8 (4.0)	74.0*** (13.7)	52.4 (18.7)	79.6*** (12.2)	56.1** (19.4)	79.3*** (16.3)	65.7*** (20.9)
Above Median, Study 2	110	126	23.3*** (16.7)	47.8 (21.5)	52.4*** (3.1)	50.6** (2.5)	75.5*** (16.1)	50.9 (19.6)	77.3*** (17.4)	52.7 (20.7)	75.2*** (22.9)	62.4*** (22.6)
*Age*												
18–29, Study 1	57	70	26.3*** (14.9)	54.7* (17.5)	52.8*** (4.1)	50.5 (3.5)	71.3*** (14.1)	52.1 (16.1)	79.3*** (13.0)	55.1* (19.1)	78.6*** (17.9)	66.0*** (21.2)
18–29, Study 2	124	136	22.6*** (16.8)	51.3 (21.9)	52.4*** (2.5)	50.5* (2.3)	72.3*** (17.5)	47.5 (20.2)	80.3*** (15.4)	53.3 (19.8)	76.9*** (22.1)	61.8*** (21.7)
30–39, Study 1	54	54	28.8*** (19.3)	56.5* (19.2)	53.3*** (4.1)	50.8 (3.9)	71.6*** (12.0)	50.0 (19.8)	80.2*** (13.7)	56.5* (19.9)	77.1*** (20.5)	62.1*** (22.5)
30–39, Study 2	91	88	24.1*** (18.6)	49.5 (22.5)	52.0*** (3.1)	50.5* (2.4)	73.4*** (17.4)	51.1 (22.0)	75.6*** (19.1)	53.1 (21.9)	73.8*** (25.3)	61.8*** (23.7)
40+, Study 1	47	40	27.2*** (16.1)	51.6 (19.6)	53.0*** (3.9)	51.2* (3.3)	73.6*** (15.9)	51.4 (20.0)	77.3*** (15.1)	55.5 (20.0)	81.2*** (13.6)	67.5*** (18.1)
40+, Study 2	54	62	25.1*** (18.3)	46.2 (24.1)	52.2*** (3.1)	50.6 (2.7)	75.1*** (15.6)	52.3 (22.1)	76.2*** (16.6)	53.1 (24.4)	77.8*** (16.8)	59.9** (25.9)

*p < .05.

**p < .01.

***p < .001.

Standard deviations appear in parentheses.

A similar pattern emerges when we examine the effect of risk-taking on trait attributions in Study 2. Once again, we find the same results as our main analysis and remarkable consistency in the effects of successful and failed risk-taking on the perception of different traits relative to risk-avoidance (see Table 11). Across all demographic groups, successful risk-takers are seen as: more likable, much more dominant, much more agentic, much less indecisive, more competent, and less foolish than risk-avoiders (Table 11). Moreover, failed risk-takers were seen across all demographic groups as more dominant, much more agentic, and much less indecisive than risk-avoiders. With the except of participants without college degrees (as the coefficient was not statistically significant, although trending in the same direction), failed risk-takers were also seen as more foolish than risk-avoiders. In sum, we again find evidence for a cultural consensus on the effect of risk-taking on attributions.

**Table 11:** Multilevel mixed models predicting trait attributions using success and failure by demographic categories (pooled scenarios).

	*Gender*		*Race*		*Education*		*Hiring*		*Management*		*Income*			*Age*		
	Man	Woman	White	Non-White	College	No College	Hiring	No Hiring	Manage	No Manage	Below Median	Median	Above Median	18–29	30–39	40+
	Coef. (RSE)	Coef. (RSE)	Coef. (RSE)	Coef. (RSE)	Coef. (RSE)	Coef. (RSE)	Coef. (RSE)	Coef. (RSE)	Coef. (RSE)	Coef. (RSE)	Coef. (RSE)	Coef. (RSE)	Coef. (RSE)	Coef. (RSE)	Coef. (RSE)	Coef. (RSE)
**Likable**																
Success	10.4*** (2.0)	15.1*** (1.8)	11.3*** (1.4)	19.1*** (3.0)	12.2*** (1.5)	15.2*** (2.5)	13.5*** (1.8)	12.7*** (1.9)	12.4*** (1.5)	14.9*** (2.6)	14.0*** (2.1)	14.1*** (3.6)	12.1*** (1.9)	18.6*** (2.0)	9.7*** (2.0)	6.3* (3.1)
Failure	3.4 (1.9)	7.4*** (1.7)	5.4*** (1.4)	5.5 (3.0)	4.9*** (1.5)	6.8** (2.4)	6.0*** (1.8)	4.8* (1.9)	4.5** (1.6)	7.3*** (2.2)	4.8* (2.1)	4.0 (3.4)	6.4*** (1.8)	7.4*** (1.9)	3.7 (2.1)	3.7 (2.9)
Intercept	49.3*** (1.3)	48.3*** (1.2)	49.8*** (1.0)	45.3*** (1.9)	48.6*** (1.0)	49.3*** (1.7)	47.2*** (1.2)	50.7*** (1.3)	49.0*** (1.0)	48.3*** (1.7)	49.7*** (1.4)	48.0*** (2.5)	48.0*** (1.3)	46.5*** (1.3)	49.6*** (1.5)	52.5*** (1.8)
N	502	608	848	262	772	338	614	496	770	340	474	162	472	520	358	232
**Dominant**																
Success	19.3*** (2.0)	23.6*** (1.8)	21.7*** (1.5)	21.9*** (2.9)	19.5*** (1.6)	26.9*** (2.4)	22.8*** (1.7)	20.5*** (2.1)	22.6*** (1.5)	19.2*** (2.7)	22.5*** (2.0)	23.1*** (4.0)	20.5*** (2.0)	21.4*** (1.9)	19.4*** (2.3)	26.3*** (3.0)
Failure	24.2*** (1.9)	23.4*** (1.7)	24.4*** (1.4)	21.6*** (2.9)	23.7*** (1.6)	24.0*** (2.3)	23.3*** (1.7)	24.4*** (2.1)	26.4*** (1.6)	18.8*** (2.3)	24.5*** (2.0)	25.6*** (3.8)	22.5*** (1.9)	19.9*** (1.9)	26.6*** (2.3)	28.2*** (2.8)
Intercept	22.9*** (1.2)	16.1*** (1.2)	19.5*** (1.0)	17.9*** (1.9)	20.1*** (1.0)	17.0*** (1.5)	19.1*** (1.1)	19.2*** (1.3)	19.0*** (1.0)	19.5*** (1.6)	18.7*** (1.3)	18.3*** (2.4)	20.0*** (1.3)	19.8*** (1.2)	19.1*** (1.5)	17.8*** (1.9)
N	502	608	848	262	772	338	614	496	770	340	474	162	472	520	358	232
**Agency**																
Success	41.8*** (2.1)	52.6*** (1.9)	47.1*** (1.6)	49.8*** (3.0)	45.9*** (1.7)	52.2*** (2.6)	48.9*** (1.9)	46.4*** (2.1)	48.0*** (1.6)	46.9*** (2.6)	47.6*** (2.2)	49.9*** (3.9)	47.1*** (2.1)	48.8*** (2.0)	46.4*** (2.5)	47.9*** (3.1)
Failure	39.5*** (2.0)	45.7*** (1.9)	43.5*** (1.5)	40.7*** (3.0)	41.6*** (1.6)	45.9*** (2.5)	43.2*** (1.8)	42.5*** (2.1)	42.6*** (1.7)	43.6*** (2.3)	42.4*** (2.2)	43.0*** (3.7)	43.5*** (2.0)	41.8*** (2.0)	43.5*** (2.5)	44.5*** (3.0)
Intercept	32.0*** (1.2)	27.5*** (1.1)	30.2*** (0.9)	27.4*** (1.7)	30.2*** (0.9)	28.0*** (1.5)	28.9*** (1.1)	30.3*** (1.2)	29.6*** (1.0)	29.3*** (1.5)	30.8*** (1.3)	29.8*** (2.2)	28.1*** (1.2)	28.9*** (1.2)	29.5*** (1.4)	31.1*** (1.8)
N	502	608	848	262	772	338	614	496	770	340	474	162	472	520	358	232
**Indecisive**																
Success	-34.3*** (2.2)	-38.6*** (1.9)	-36.6*** (1.7)	-37.0*** (2.9)	-36.6*** (1.7)	-37.1*** (2.6)	-35.0*** (2.0)	-38.9*** (2.1)	-36.8*** (1.7)	-36.7*** (2.7)	-36.9*** (2.2)	-39.6*** (3.7)	-35.6*** (2.2)	-37.3*** (2.0)	-34.4*** (2.6)	-39.5*** (3.2)
Failure	-27.0*** (2.1)	-31.6*** (1.9)	-29.5*** (1.6)	-28.9*** (2.9)	-28.4*** (1.7)	-31.6*** (2.5)	-28.7*** (1.9)	-30.2*** (2.1)	-29.7*** (1.8)	-28.7*** (2.4)	-30.5*** (2.2)	-27.6*** (3.6)	-29.0*** (2.1)	-30.5*** (2.0)	-27.5*** (2.6)	-29.4*** (3.0)
Intercept	49.9*** (1.3)	48.0*** (1.1)	49.1*** (1.0)	47.9*** (1.7)	49.1*** (1.0)	48.3*** (1.5)	48.3*** (1.1)	49.6*** (1.2)	49.3*** (1.0)	47.9*** (1.5)	49.1*** (1.3)	48.2*** (2.1)	48.9*** (1.3)	49.1*** (1.2)	47.5*** (1.5)	50.3*** (1.8)
N	502	608	848	262	772	338	614	496	770	340	474	162	472	520	358	232
**Competence**																
Success	16.7*** (2.1)	24.3*** (2.0)	19.0*** (1.6)	27.6*** (3.2)	20.5*** (1.6)	22.1*** (2.9)	21.8*** (2.0)	20.1*** (2.0)	21.3*** (1.7)	19.9*** (2.7)	20.5*** (2.3)	22.6*** (4.0)	21.1*** (2.0)	23.9*** (2.1)	19.4*** (2.5)	17.2*** (3.2)
Failure	0.5 (2.0)	5.6** (1.9)	2.1 (1.6)	6.4* (3.2)	1.7 (1.6)	6.5* (2.8)	4.1* (1.9)	1.9 (2.0)	2.1 (1.7)	5.4* (2.4)	2.1 (2.4)	5.0 (3.8)	3.5 (1.9)	4.1* (2.0)	3.3 (2.6)	0.7 (3.1)
Intercept	55.9*** (1.3)	54.4*** (1.3)	56.7*** (1.0)	49.7*** (2.0)	55.1*** (1.0)	54.9*** (1.8)	54.4*** (1.2)	55.9*** (1.3)	55.2*** (1.1)	54.6*** (1.7)	56.9*** (1.4)	55.5*** (2.5)	52.9*** (1.3)	53.2*** (1.3)	55.8*** (1.6)	58.0*** (2.0)
N	502	608	848	262	772	338	614	496	770	340	474	162	472	520	358	232
**Foolishness**																
Success	-10.7*** (2.3)	-16.2*** (2.0)	-12.7*** (1.8)	-17.7*** (3.0)	-12.8*** (1.9)	-16.2*** (2.8)	-12.8*** (2.1)	-15.0*** (2.2)	-13.1*** (1.9)	-15.9*** (2.8)	-14.4*** (2.4)	-13.6*** (4.0)	-13.4*** (2.3)	-16.3*** (2.1)	-9.3*** (2.7)	-16.6*** (3.6)
Failure	12.1*** (2.2)	6.1** (2.0)	9.7*** (1.7)	6.5* (3.1)	10.8*** (1.8)	4.8 (2.7)	7.2*** (2.1)	11.1*** (2.2)	10.0*** (1.9)	6.9** (2.5)	10.1*** (2.4)	9.1* (3.8)	7.6*** (2.2)	7.3*** (2.1)	12.0*** (2.8)	8.9** (3.4)
Intercept	31.1*** (1.3)	28.6*** (1.2)	29.6*** (1.0)	30.3*** (1.9)	29.7*** (1.1)	29.9*** (1.6)	30.6*** (1.2)	28.6*** (1.3)	30.2*** (1.1)	28.7*** (1.6)	29.3*** (1.4)	30.2*** (2.3)	30.1*** (1.4)	30.2*** (1.3)	27.0*** (1.6)	32.8*** (2.0)
N	502	608	848	262	772	338	614	496	770	340	474	162	472	520	358	232

*p < .05.

**p < .01.

***p < .001.

Robust standard errors appear in parentheses.

## Discussion and conclusions

Existing literature on negativity bias and the societal value of risk-taking provide competing hypotheses for how workplace risk-takers will be understood by observers. The data here are the first (to our knowledge) that quantitatively measure these effects. We find observers believe that, relative to risk-avoidance: 1) successful risk-taking will improve workplace outcomes and, 2) failed risk-taking is not particularly disadvantaging at work. While we find some evidence that failed risk-takers are perceived as being more likely to be downsized, we also find that failed risk-takers are seen as more likely to be hired and promoted (relative to risk-avoiders). Mediation analyses reveal this is primarily because risk-taking—regardless of outcome—greatly increases perceptions of agency and decreases perceptions of indecisiveness, and these attributions significantly impact perceived workplace outcomes. In addition, we find evidence that there is a cultural consensus about the value of risk-taking, as our results are similar across varying participant characteristics. Thus, our results generally support the idea that risk-taking, even when resulting in failure, carries a cultural cachet that protects workers from punishment.

This research makes five core contributions. First, it expands our knowledge of how workplace risk-taking behavior influences attributions about workers. While there is ample knowledge about risk-preferences and risk-taking behavior [28], there is no quantitative research (to our knowledge) that examines how observers interpret others’ risk-taking behavior. Although qualitative research suggests that workplace risk-taking is valued in the U.S. [1,2], no research evaluates how workplace risk-taking—and its resultant outcomes—influence attributions relative to risk-avoidance. Second, these results have implications for macro-level economic conditions. Macro-level beliefs about the associated meanings—and the resulting societal value—of risk-taking behavior are likely to influence micro-level decisions about taking risks at work. In turn, these micro-level decisions can impact macro-level economic conditions (for instance, the financial crisis of 2008). Third, these results provide insight on the potential costs and benefits of risk-seeking strategies advocated in popular culture, as cultural attitudes and attributions about risk-taking are antecedents to obtaining material rewards and advancing in rank [29–31]. Fourth, these results provide new insights on the connection between attributions and workplace outcomes. We find that negative traits (foolishness, indecisiveness) are predictive of negative outcomes (downsizing), and that positive traits (agency, competence, and likability) are predictive of positive outcomes (hiring, promotion). In other words, it is not a lack of a positive quality (e.g., competence) that gets one fired: it is the perception that a worker has a negative quality (e.g., foolishness) (and vice-versa for positive outcomes). And lastly, these results have implications for—and raise important questions about—how workplace inequalities are reified and justified. We find that there are large benefits associated with successfully taking risks at work; however, not all groups of people are equally likely to be given the opportunity to take risks by those in power [18]. Moreover, different groups may experience different cost-benefit ratios from taking risks. Future research could empirically investigate these connections.

Of course, our findings have limitations. Our results are best understood as reflecting the cultural image of employees who take or do not take risks. Future research should investigate how real-world employers and co-workers act upon these attributions when evaluating their risk-taking and risk-avoidant colleagues. While our results can speak to the general cultural beliefs about risk-takers and risk-avoiders, they may not extend to extremely risk-averse or extremely risk-seeking occupations and workplaces (e.g., investors vs. fry cooks). Future research should also investigate general cultural beliefs about risk-takers and risk-avoiders in other cultural contexts, such as countries that are notoriously more averse to risk (e.g., Japan [32]), as this would give greater insight on the connection between cultural beliefs about risk-taking, workplace outcomes, and attributions about risk-takers.

Nevertheless, these findings provide important insights about how workplace risk-taking is understood by observers, leading us to conclude that, as far as attributions are concerned, workplace risk-taking is not as risky as one might think.

## Supporting information

S1 Data Part A—Full scenario text. (DOCX)

S2 Data Part B—Construction of trait indexes. (DOCX)

S1 Table Unrefined path model coefficients predicting trait attributions and workplace rewards (N = 1,110, clustered within 555 individuals). (DOCX)
