# Supplementary material for: Bold or reckless? The impact of workplace risk-taking on attributions and expected outcomes
Source: PLoS One. 2020 Mar 4;15(3):e0228672. doi: 10.1371/journal.pone.0228672 (PMC7055845; doi:10.1371/journal.pone.0228672)
Supplement: S1 Data — (DOCX) [file pone.0228672.s001.docx]

**Supporting information**

**Part A – full scenario text
Vignette 1: Suggestion at a meeting, success**Employee A and Employee B attend a brainstorming meeting at their workplace. At these sorts of meetings, their boss appreciates it when employees contribute good ideas, but hates it when employees waste time with bad ideas.

Employee A offers a suggestion which the boss deems to be a good idea. Employee B says nothing at the meeting.

**Vignette 2: Suggestion at a meeting, failure**
Employee A and Employee B attend a brainstorming meeting at their workplace. At these sorts of meetings, their boss appreciates it when employees contribute good ideas, but hates it when employees waste time with bad ideas.

Employee A offers a suggestion which the boss deems to be a bad idea. Employee B says nothing at the meeting.

**Vignette 3: Job innovation, success**
Employee A and Employee B perform the same job. Their job is routine and mundane. Both Employee A and Employee B have thought of a way to change the way that they complete the job that would theoretically increase productivity. However, there is a chance that this change would not work and actually would waste a great deal of time and energy.

Employee A does implement the change and it is a success. Employee B does not implement the change.

**Vignette 4: Job innovation, failure**Employee A and Employee B perform the same job. Their job is routine and mundane. Both Employee A and Employee B have thought of a way to change the way that they complete the job that would theoretically increase productivity. However, there is a chance that this change would not work and actually would waste a great deal of time and energy.

Employee A does implement the change and it is a failure. Employee B does not implement the change.

**Vignette 5: Leading a new initiative, success**
Employee A and Employee B are both mid-level managers at a corporation. Both have been given the opportunity to head up a major new initiative at work. Leading a major initiative like this can be a career making action for a mid-level manager if the initiative is a success. However, if the initiative is a failure for any reason, it can have disastrous, long-term career effects.

Employee A does accept the opportunity to lead the new initiative and the initiative is a success. Employee B does not accept the opportunity to lead the new initiative.

**Vignette 6: Leading a new initiative, failure**
Employee A and Employee B are both mid-level managers at a corporation. Both have been given the opportunity to head up a major new initiative at work. Leading a major initiative like this can be a career making action for a mid-level manager if the initiative is a success. However, if the initiative is a failure for any reason, it can have disastrous, long-term career effects.

Employee A does accept the opportunity to lead the new initiative and the initiative is a failure. Employee B does not accept the opportunity to lead the new initiative.
