# Supplementary material for: Bold or reckless? The impact of workplace risk-taking on attributions and expected outcomes
Source: PLoS One. 2020 Mar 4;15(3):e0228672. doi: 10.1371/journal.pone.0228672 (PMC7055845; doi:10.1371/journal.pone.0228672)
Supplement: S2 Data — (DOCX) [file pone.0228672.s002.docx]

**Part B – construction of trait indexes**Participants in Study 2 were given a list of traits, and were asked to rate each employee on each trait using a 100-point slider scale (in which 0 = “Not at all,” and 100 = “Completely”). Traits were combined to create indexes: six were measures of *likability* (“Popular with colleagues,” “Likeable,” “Someone you would want to get to know better,” “Friendly,” “Warm,” “Humble,”) (⍺ = .88), three were measures of being *domineering* (“Controlling,” “Dominating,” “Arrogant,”) (⍺ = .86), seven were measures of *workplace agency* (“Assertive,” “Driven,” “Ambitious,” “Leadership ability,” “Career-oriented,” “Hardworking,” “Self-starter,”) (⍺ = .96), three were measures of *indecisiveness* (“Uncertain,” “Indecisive,” “Weak,”) (⍺ = .86), four were measures of *workplace competence* (“Competent,” “Has necessary skills for job,” “Business sense,” “Intelligent,”) (⍺ = .91), and two were measures of *workplace foolishness* (“Poor decision- maker,” “Foolish,”) (*r* = .63). Participants were also asked to independently rate each employee on three measures of workplace outcomes using the 100-point slider scales (in which 0 = “Not at all,” and 100 = “Completely”): “Someone you would downsize,” “Someone you would choose to interview,” and “Someone you would choose to promote.” We included these independent ratings of workplace outcomes for each employee, in addition to the fixed pool of 100 percentage points distributed between the two employees, in order to have independent ratings the risk- taking and risk-avoiding employee for the mediation analysis.
