# Supplementary material for: Bold or reckless? The impact of workplace risk-taking on attributions and expected outcomes
Source: PLoS One. 2020 Mar 4;15(3):e0228672. doi: 10.1371/journal.pone.0228672 (PMC7055845; doi:10.1371/journal.pone.0228672)
Supplement: S1 Table — (DOCX) [file pone.0228672.s003.docx]

| **Table S1. Unrefined Path Model Coefficients Predicting Trait Attributions and Workplace Rewards (N = 1,110, Clustered within 555 Individuals).** | | | | | | | | | | |
| --- | --- | --- | --- | --- | --- | --- | --- | --- | --- | --- |
|  | *Trait Attributions* | | | | | |  | *Workplace Rewards* | | |
|  | Indecisive | Foolish | Competent | Dominant | Agentic | Likable |  | Promote | Interview | Downsize |
|  | Coef.  (Robust SE) | Coef.  (Robust SE) | Coef.  (Robust SE) | Coef.  (Robust SE) | Coef.  (Robust SE) | Coef.  (Robust SE) |  | Coef.  (Robust SE) | Coef.  (Robust SE) | Coef.  (Robust SE) |
| Successful Risk-Taking | -36.73***  (1.33) | -13.77***  (1.42) | 21.06***  (1.42) | 21.28***  (1.44) | 47.78***  (1.42) | 13.46***  (1.38) |  | 7.45***  (1.82) | 1.13  (1.66) | -5.74*  (2.37) |
| Failed  Risk-Taking | -29.40***  (1.37) | 8.87***  (1.64) | 3.13*  (1.38) | 24.21***  (1.37) | 42.88***  (1.35) | 5.14***  (1.26) |  | 1.29  (1.81) | 2.04  (1.73) | -3.24  (2.33) |
| Indecisive |  |  |  |  |  |  |  | .01  (.03) | .01  (.03) | .19***  (.04) |
| Foolish |  |  |  |  |  |  |  | -.08*  (.03) | -.10**  (.04) | .38***  (.05) |
| Dominant |  |  |  |  |  |  |  | -.01  (.03) | .00  (.03) | .13***  (.04) |
| Competent |  |  |  |  |  |  |  | .41***  (.04) | .43***  (.05) | -.21***  (.06) |
| Agentic |  |  |  |  |  |  |  | .38***  (.04) | .27***  (.04) | -.13**  (.05) |
| Likable |  |  |  |  |  |  |  | .26***  (.04) | .38***  (.05) | .04  (.06) |
| Constant | 48.86***  (.97) | 29.73***  (.89) | 55.06***  (.97) | 19.15***  (.76) | 29.53***  (.89) | 48.77***  (.90) |  | -6.72***  (1.86) | -.92  (2.01) | 29.27***  (3.40) |
| *NOTE*: Fit statistics: *χ^2^* = 2,066.02, *df* = 18, *p* < .001; CFI = .679; TLI = .037; RMSEA = .320; SRMR = .181. **p* < .05. ***p* < .01. ****p* < .001. | | | | | | | | | | |
